# Metastatic Melanoma to Ovary and Advances in Metastatic Melanoma Treatment: A Case Report

**DOI:** 10.7759/cureus.77664

**Published:** 2025-01-19

**Authors:** Melissa Cantave, Anita H Chen, Aakriti R Carrubba, Matthew Robertson

**Affiliations:** 1 Department of Medical and Surgical Gynecology, Mayo Clinic, Jacksonville, USA

**Keywords:** adnexal mass, genetic testing, gynecologic surgery, immunotherapy, metastatic melanoma

## Abstract

Metastatic lesions to the ovary from melanoma are very rare. There have been several published case reports over the years documenting melanoma metastasizing to the adnexa. Ovarian metastases may either be asymptomatic or present with pelvic pain. As with most adnexal masses, diagnosis can be challenging and depends on the quality of imaging. When present, the prognosis is very poor. Surgical excision can be offered for symptomatic management, confirmation or diagnosis, and for cytoreduction; however, advances in genetic testing and systemic therapy have shown promise in treating this disease. To add to the growing body of literature, we present a case of melanoma metastasizing to the ovary and will discuss the advances in systemic therapy as well as genetic testing for these patients.

## Introduction

Melanoma can metastasize to unique locations in the body, and metastatic lesions to the ovary are rare. Over the years, there have been several published case reports documenting melanoma metastasizing to the adnexa [1-3]. When present, the prognosis is very poor with a survival rate of around 5%-7% at five years [1]. Past guidelines recommend surgical management with total abdominal hysterectomy and bilateral salpingo-oophrectomy [3]. Advances in genetic testing and systemic therapy including immune checkpoint inhibitors have shown promise in not only developing target therapies but also improving survival for patients with metastatic melanoma. To add to the growing body of literature, we present a case of melanoma metastasizing to the ovary and will discuss the advances in systemic therapy as well as genetic testing for these patients.

## Case presentation

**Figure 1:**
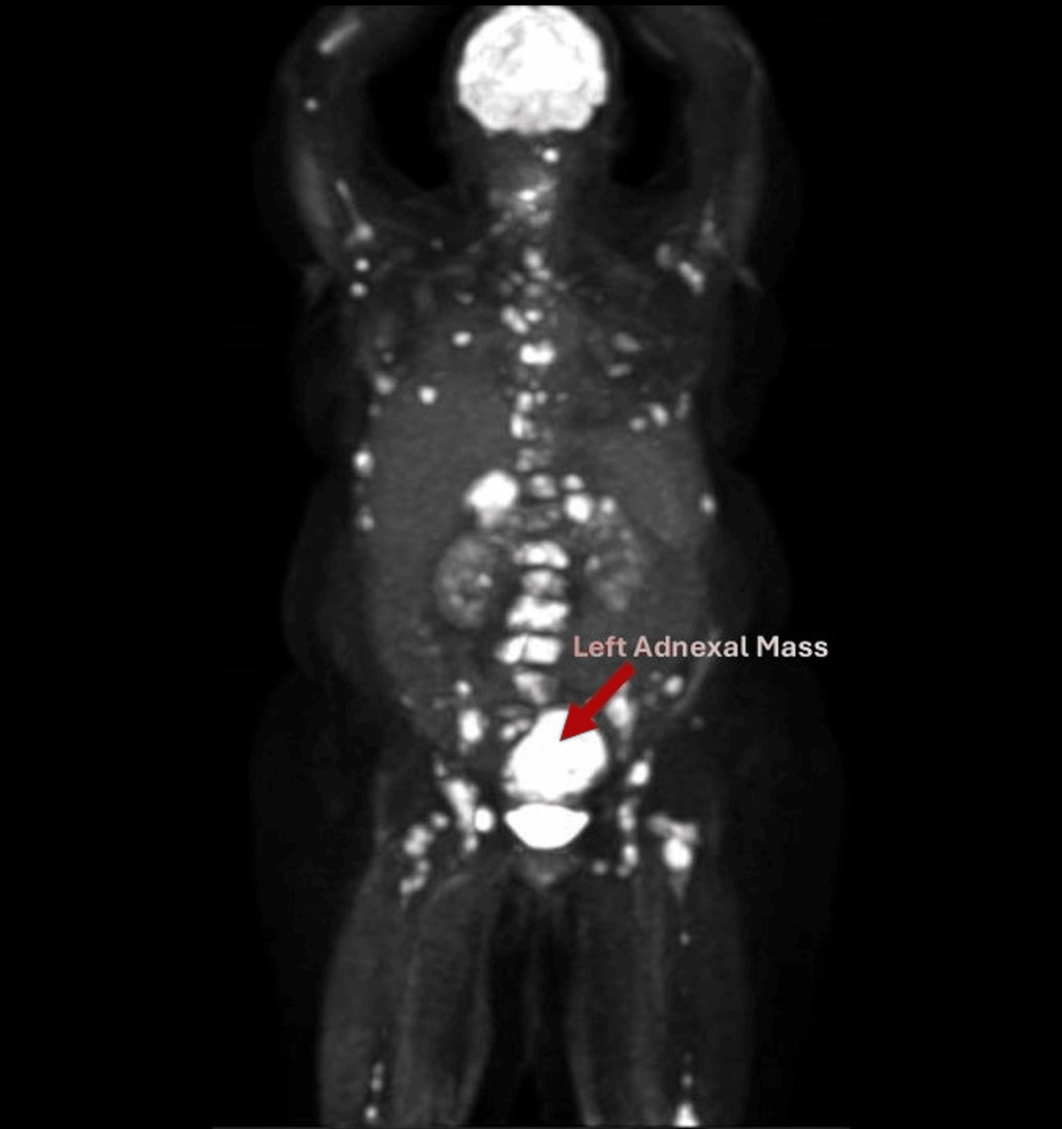
CT-PET image (sagittal view) CT-PET: computed tomography-positron emission tomography PET maximum intensity image on the left illustrates widespread metastases of the malignant melanoma, including the left adnexal mass pictured above

**Figure 2:**
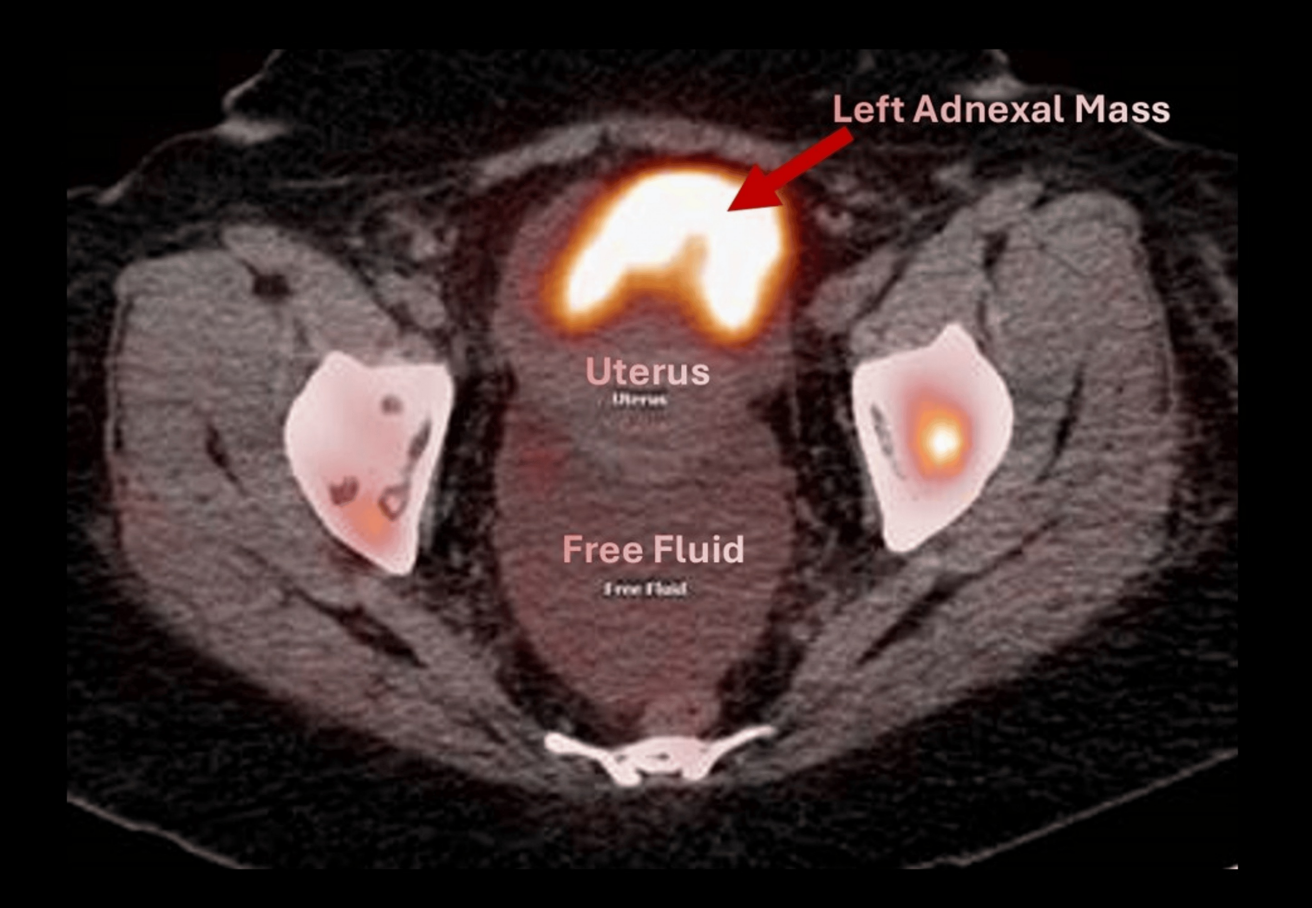
CT-PET image (axial view) CT-PET: computed tomography-positron emission tomography The overlay PET/CT scan on the right shows the left adnexal mass as well as free fluid in the pelvis in the axial view

A 48-year-old G2P2 patient presented to the gynecology service as a referral for management of an adnexal mass. She had previously presented to an outside hospital emergency room with left flank pain and hematuria. Her past medical history is significant for a superficial spreading melanoma excised from her back in 2016. She was recommended to have surveillance exams with dermatology; however, she was not adherent. Ultimately, she was lost to follow-up and presented eight years later with flank pain. Outside hospital imaging revealed a 6.7 cm left adnexal mass, 2.7 cm bladder mass, extensive osseous metastatic disease, and metastatic adrenal nodules with moderate to severe ascites (Figures 1-2). She was ultimately referred to Gynecologic Oncology for further management of her adnexal mass. An abdominal exam in the office was notable for a distended abdomen with a positive fluid wave. 

**Figure 3:**
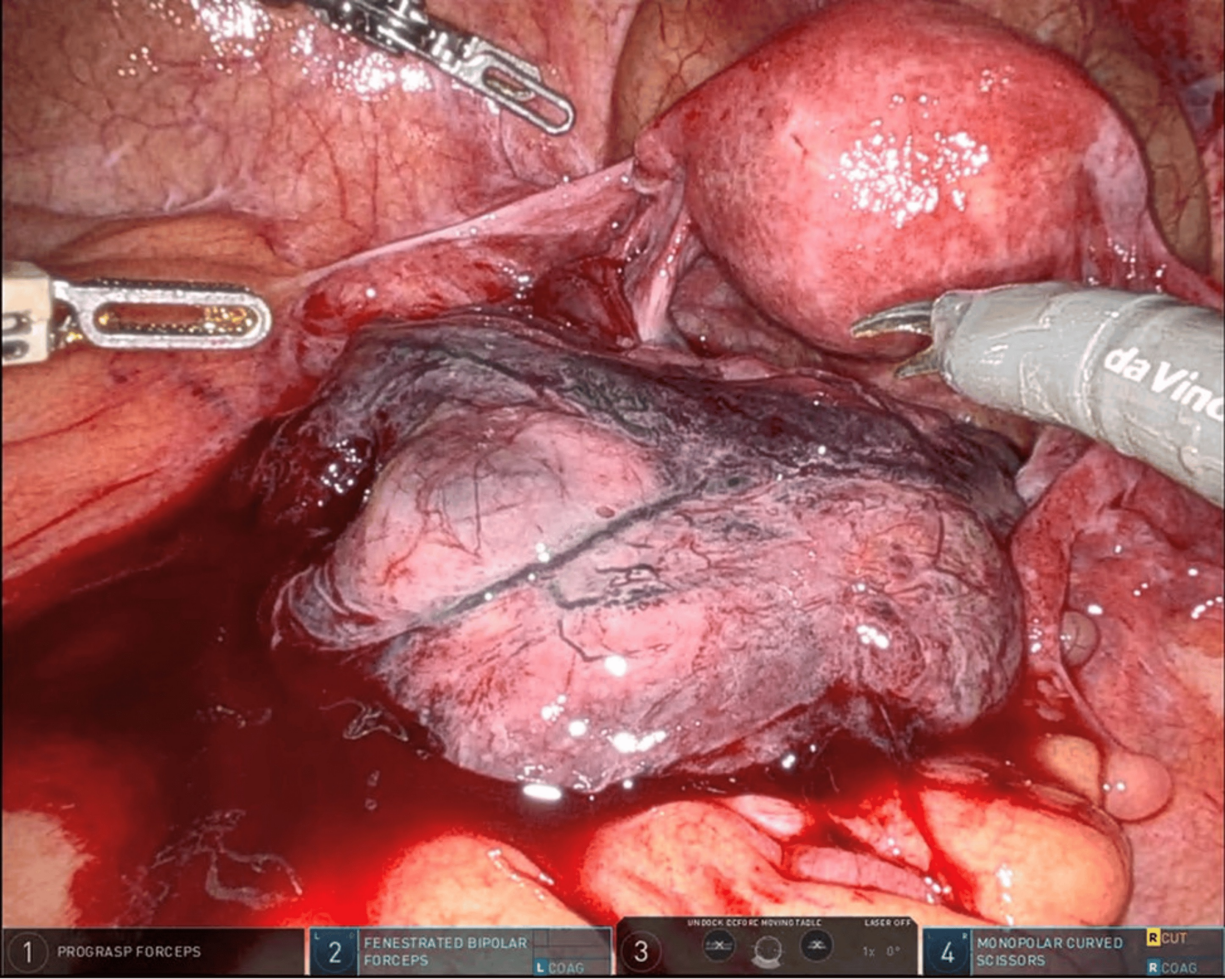
Intraoperative laparoscopy image On laparoscopy, the left adnexa was enlarged and edematous. Copious bloody ascites was evacuated prior to hysterectomy

She underwent a robotic-assisted total laparoscopic hysterectomy and bilateral salpingo-oophrectomy in order to debulk abdominal disease. Intraoperative laparoscopy images demonstrated an enlarged, blackened left adnexa (Figure 3). Copious bloody ascites were noted upon entry and evacuated prior to hysterectomy. Intraoperative frozen pathology revealed metastatic melanoma. 

**Figure 4:**
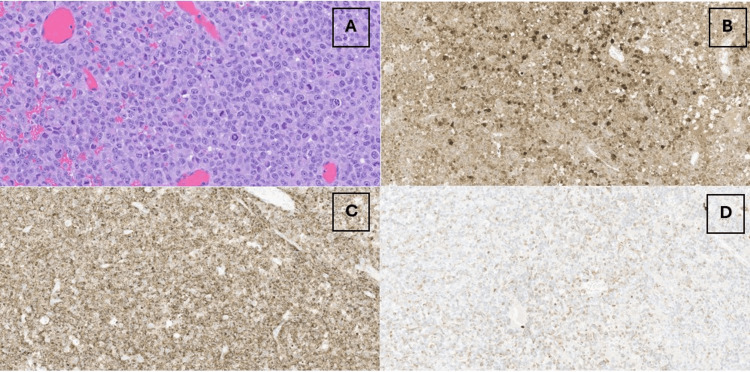
Pathology of the left ovarian tumor The left ovarian tumor was composed of pattern-less sheets of atypical polygonal cells with prominent nucleoli and numerous mitotic figures on hematoxylin and eosin (H&E) slide as seen throughout the entire slide (A). This image is set at 320X (A). Immunostains are strongly positive for S100 (B) and HMB45 (C), set at 130X. Aberrant keratin expression is apparent on pathology (D), which is also set at 130X. Aberrant keratin expression is well-known in melanoma

Final histopathology confirmed metastatic melanoma involving both ovaries (Figure 4). Pelvic washings were significant for rare, atypical mesothelial cells. The uterus, cervix, and bilateral fallopian tubes were uninvolved by the tumor. Immunostains were strongly positive for S100 and HMB45 and were weakly focally positive for cytokeratin. She recovered well from the procedure and was referred to Clinical Genomics for genetic testing as well as her local medical oncologist for further treatment. She completed a comprehensive hereditary genetic testing panel that tested for numerous gene mutations associated with melanoma including CDK4 and BAP1, and her genetic testing demonstrated no pathologic mutations. She started on nivolumab and ipilimumab for systematic treatment and is being followed by her local medical oncologist. 

## Discussion

Melanoma can be unpredictable in its disease spread. More commonly, melanoma can spread to the lungs, brain, liver, eyes, kidney, or bone. Prior case reports have been published documenting the novelty of metastatic lesions to the ovary [1-2]. There are some case reports documenting similar cases of melanoma presenting as metastatic years after initial diagnosis [1-2]. Our case shows the novel course of melanoma presenting years later with widespread disease noted in the ovaries, adrenal glands, and bone metastases. Ovarian metastases may either be asymptomatic or present with pelvic pain, such as in this patient. It can be challenging to differentiate primary ovarian malignancies from adnexal metastasis on imaging modalities such as ultrasound or CT scan, and immunohistochemical stains can provide definitive diagnosis [4]. S-100 is one of the most sensitive markers for melanoma; in addition, HMB-45 has been shown to be associated with melanosome production, which is seen in malignant melanoma [5-6].

Genetic testing has now proven to be beneficial for developing targeted cancer treatments. Genetic mutations associated with metastatic melanoma include B-Raf proto-oncogene (BRAF) mutations, specifically V600E mutations [7]. BRAF mutations are present in approximately 40%-50% of melanomas, and these mutations lead to constitutional activation of the MAP-K/EPK signaling pathway, which promotes cell proliferation and survival [7]. Other less common BRAF mutations include V600K, V600R, and non-V600 mutations [7]. The National Comprehensive Cancer Network (NCCN) as well as the American Society of Clinical Oncology recommend genetic testing for patients with unresectable or metastatic melanoma to guide treatment, especially if patients are found to be BRAF mutation carriers [8-9].

Additional genetic testing on tumor specimens aids in tailoring systematic treatment. Our pathology department performed additional genetic testing on the patient’s tumor, and two clinically relevant variants were detected. First, the patient’s tumor tested positive for a telomerase reverse transcriptase (TERT) mutation, specifically the C228T variant. TERT encodes telomerase reverse transcriptase protein, leading to increased telomerase activity, protein expression, and increased transcriptional activity [10]. TERT can be activated through multiple mechanisms in several cancer types including primary glioblastoma, bladder, and hepatocellular cancers [11]. TERT promotor mutations have been reported in approximately 22%-68% of melanoma cases and have been shown to be an adverse prognostic indicator [10,12]. Second, our patient’s tumor profile was significant for a neuroblastoma rat sarcoma viral oncogene homolog. NRAS encodes a specific protein in the family of Ras proteins, which mediates the transduction of growth signals [13]. Activating NRAS mutations produces oncogenic activation of downstream signaling pathways including Raf/MEK/ERK, causing cell growth, differentiation, and survival [13]. Just as with TERT-promoting mutations, activating NRAS mutations is associated with poor prognosis [14].

Prior to the advent of immune checkpoint inhibitors and modulators, metastatic ovarian melanoma was managed surgically with total abdominal hysterectomy with bilateral salpingo-oophrectomy [3]. Surgical excision can be offered for symptomatic management of pain, confirmation of diagnosis, or cytoreduction [3]. Systemic therapy is required for definitive cancer management. Targeted immunotherapy based on identifiable pathologic mutations such as BRAF V600 mutations has improved survival for patients with metastatic melanoma. Current cornerstone treatments for metastatic melanoma include immune checkpoint modulators, such as LAG3 inhibitors, pembrolizumab, nivolumab, and ipilimumab targeting CTLA-4, and PDL1 [9,15]. Nivolumab is a monoclonal antibody that targets PD1 and is used in combination with ipilimumab, which targets cytotoxic T-lymphocyte antigen-4 (CTLA-4) [16-18]. If BRAF mutation is present, utilizing BRAF inhibitors such as dabrafenib and vemurafenib and MEK inhibitors such as trametinib is the first-line treatment as research studies have shown that these treatments improve progression-free survival [19]. Mitogen-activated protein kinase (MEK) protein is critical in the RAS-RAF-MEK signaling pathway, and dysregulation has been seen in various cancer types like melanoma. Some investigative trials have shown that NRAS activating mutations may predict response to certain MEK inhibitors [16,20].

## Conclusions

This case reflects the unique changes and advances in the management and treatment of metastatic melanoma. Prior to the advent of immunotherapy and targeted genetic testing, patients with metastatic melanoma would undergo surgical management with abdominal hysterectomy with bilateral salpingo-oophrectomy. Now, treatment guidelines have been updated to reflect the use of targeted immunotherapy to treat and improve patient outcomes for metastatic melanoma. While surgical excision can be offered for symptomatic management of abdominal or pelvic pain, confirmation of diagnosis, and cytoreduction, systematic therapy is often required for cancer treatment. Advances in genetic testing have made way for the development of novel targeted therapies based on oncogenes specific to melanoma. 
